# Childhood Cancer Incidence in British Indians & Whites in Leicester, 1996–2008

**DOI:** 10.1371/journal.pone.0061881

**Published:** 2013-04-17

**Authors:** Shameq Sayeed, Isobel Barnes, Benjamin J. Cairns, Alexander Finlayson, Raghib Ali

**Affiliations:** 1 Cancer Epidemiology Unit, University of Oxford, Richard Doll Building, Oxford, United Kingdom; 2 INDOX Cancer Research Network, Cancer Epidemiology Unit, Richard Doll Building, Oxford, United Kingdom; 3 Faculty of Medicine and Health Sciences, United Arab Emirates University, Al-Ain, United Arab Emirates; Tehran University of Medical Sciences, Iran (Islamic Republic of)

## Abstract

**Background:**

South Asians in England have an increased risk of childhood cancer but incidence by their individual ethnicities using self-assigned ethnicity is unknown. Our objective was to compare the incidence of childhood cancer in British Indians and Whites in Leicester, which has virtually complete, self-assigned, ethnicity data and the largest population of Indians in England.

**Methods:**

We obtained data on all cancer registrations from 1996 to 2008 for Leicester with ethnicity obtained by linkage to the Hospital Episodes Statistics database. Age-standardised incidence rates were calculated for childhood cancers in Indians and Whites as well as rate ratios, adjusted for age.

**Results:**

There were 33 cancers registered among Indian children and 39 among White children. The incidence rate for Indians was greater compared to Whites for all cancers combined (RR 1.82 (95% CI 1.14 to 2.89); p = 0.01), with some evidence of increased risk of leukaemia (RR 2.20 (0.95 to 5.07); p = 0.07), lymphoma (RR 3.96 (0.99 to 15.84); p = 0.04) and central nervous system tumours (RR 2.70 (1.00 to 7.26); p = 0.05). Rates were also higher in British Indian children compared to children in India.

**Conclusions:**

British Indian children in Leicester had an increased risk of developing cancer compared to White children, largely due to a higher incidence of central nervous system and haematological malignancies.

## Introduction

Childhood cancer is the second most common cause of death in children (aged 0–14) in the UK [1] with evidence of increasing incidence of leukaemias and lymphomas [2]–[4]. Yet despite major advances in their diagnosis and treatment, little is known regarding the aetiology of these cancers. Differences between ethnic groups can provide clues about possible risk factors thus potentially benefiting both the ethnic groups and the wider population [5], [6]. In contrast to studies of cancer incidence in South Asian adults (which have shown a decreased risk for many cancers, compared to non-South Asians) [7]–[10], studies of childhood cancer have suggested there is either a similar or increased (and overall possibly increasing annual) risk in South Asian children compared to their non-South Asian counterparts, particularly for leukaemias and lymphomas [11]–[13]. However, South Asians are a heterogeneous group with widely varied backgrounds and socio-cultural practices and the risk of childhood cancer by their individual self-assigned ethnicities (Indian, Pakistani and Bangladeshi) is unknown. Since 1995, however, self-assigned ethnicity has been recorded in the Hospital Episodes Statistics (HES) database (using the same classification as the Census), and HES data can now be linked to cancer registration data, so providing more reliable information on ethnicity and allowing individual ethnic groups to be analysed separately [14], [15].

British Indians are the largest ethnic minority group in the UK, with more than one million people identifying themselves as Indian in the 2001 UK Census. Leicester was chosen for this analysis because it has virtually complete self-assigned ethnicity data for each cancer registration [9] and is home to the largest population of British Indians of any local authority in the UK. We have previously shown how cancer incidence in British Indian and White adults varies in Leicester [9] and here we present our findings for cancer incidence in children.

## Patients and Methods

### Data collection

We obtained data from the Trent Cancer Registry for all cancer registrations from January 1996 to December 2008 in residents of Leicester aged 0–14 years old. For each registration the following information was given: cancer site coded to the International Classifications of Diseases, 10^th^ Revision (ICD-10) [16]; age at diagnosis of cancer; and, self-assigned ethnicity from linked records in the Hospital Episode Statistics (HES) database.

For the years 2001–2007, we were able to use mid-year population estimates, stratified by age and ethnicity, which are produced by the Office of National Statistics (ONS) [17]. However, for the years 1996–2000 (where ONS have not produced such estimates), we calculated population estimates, stratified by age and ethnicity using data provided by the ONS as follows: we linearly interpolated the distributions of ethnicity in Leicester for each year using population data from the 1991 and 2001 Census and then applied these distributions to mid-year population estimates, stratified by age.

### Classification of cancers

Cancers were classified as leukaemia (ICD-10 codes C91–95); lymphoma (C81–C85); central nervous system (CNS) tumours (ICD-10 codes C70–C72); we also examined all other solid tumours and all cancers.

We further arranged the cancers into 4 subgroups (leukaemia, lymphoma, CNS tumours, all other solid tumours) based upon the International Classification of Childhood Cancer (ICCC-3) system [18], corresponding respectively to its diagnostic groups I, II, III and IV–XII, as used in previous studies [11].

### Classification of ethnicity

Prior to April 2001, ethnicity was classified by HES according to the codes used in the 1991 Census. After April 2001, the codes were amended to conform to those used in the 2001 Census. For the analyses presented in this paper, we have classified ethnicity as White (White from the 1991 Census and British White, Irish White and Other White from the 2001 Census) and Indian (Indian from the 1991 and 2001 Censuses).

### Statistical analyses

We estimated age standardised incidence rates (ASRs) of cancer per 100 000 person years and their 95% confidence intervals (95% CIs) using direct standardisation to the 1960 Segi world population [19].

We estimated rate ratios (RRs) comparing the incidence of cancer in British Indians with that in Whites and their 95% CIs using Poisson regression, adjusted for age (0–4, 5–9 and 10–14 years). We tested the statistical significance of ethnicity using log-likelihood ratio chi-square tests. We performed goodness-of-fit chi-square tests that showed no real evidence to suggest that the data did not fit the Poisson models (all p>0.36).

To assess the effect of missing ethnicity information (7 cases (7.4%)), we performed a sensitivity analysis using multiple imputations of the missing ethnicity values based on age, sex, and site of cancer. Analysis was restricted to all cancers due to the small number of cases with missing ethnicity.

We conducted all analyses using the STATA software package (release 11).

### Comparison of British Indian children to children in India

We obtained age standardised incidence rates for children in India from *Cancer Incidence in Five Continents (CI5)* [20], [21] which are also standardised to the 1960 Segi world population. As the majority of Indians in Leicester are of Gujarati origin (approximately 70%) [22], we compared our results to those from cancer registries in Ahmedabad (capital of Gujarat) (1993–1997) and Mumbai (1998–2002), the latter having the largest Gujarati population in India outside Gujarat [23].

### Ethics statement

This study was approved by the Oxford Research Ethics Committee.

## Results

Demographic information from the 2001 Census for Indian and White children in Leicester is presented in Table 1. There were just over half as many Indian children aged 0–14 (n = 17414) as White children (n = 31896). They were similar with regards to distribution by sex, age and socioeconomic status.

**Table 1:** Comparison of demographics for British Indian and British White children living in Leicester and for children living in Mumbai and Ahmedabad (data from the 2001 Census of England and Wales and the 2001 Census of India).

		Leicester						India			
		British Indians		British Whites		Other Ethnicities		Mumbai		Ahmedabad	
		N	(%)	N	(%)	N	(%)	N	(%)	N	(%)
Total population		17 414	(100.0)	31 896	(100.0)	9 043	(100.0)	3 180 084	(100.0)	1 742 337	(100.0)
Sex	Male	8 920	(51.2)	16 152	(50.6)	4 556	(50.4)	1 648 695	(51.8)	945 167	(54.2)
Age	<5	5 355	(30.8)	10 596	(33.2)	3 167	(35.0)	949 092	(29.8)	531 956	(30.5)
	5–9	5 809	(33.4)	10 651	(33.4)	3 023	(33.4)	1 041 038	(32.7)	588 873	(33.8)
	10–14	6 250	(35.9)	10 649	(33.4)	2 853	(31.6)	1 149 648	(36.2)	621 508	(35.7)
Deprivation*	Quintile 1 (high deprivation)	10 652	(61.2)	18 275	(57.3)	5 961	(65.9)				
	Quintile 2	4 092	(23.5)	6 190	(23.5)	1 767	(19.5)				
	Quintile 3	1 977	(11.4)	4 497	(11.4)	911	(10.1)				
	Quintile 4	520	(3.0)	2 167	(6.8)	328	(3.6)				
	Quintile 5 (low deprivation)	173	(1.0)	767	(2.4)	76	(0.8)				

* Deprivation assessed from national quintiles of the income domain of the Multiple Index of Deprivation 2007 [40].

There were a total of 94 cancer cases registered from Jan 1996–Dec 2008, of which 39 (41.5%) were in White, 33 (35.1%) in Indian, and 15 (16.0%) in other ethnic group children. Data on site of cancer, age and sex were complete, but ethnicity data was missing in 7 cases (i.e. 92.6% complete).

### Comparison of Indian to White children in Leicester

The numbers of cases of cancers and estimates of the age standardised incidence rates in White and Indian children are presented in Table 2. The three most common cancers in Indians were leukaemia (n = 11), CNS tumours (n = 9), and lymphoma (n = 6), while the three most common in Whites were leukaemia (n = 11), bone, connective and soft tissue cancers (n = 8), and CNS tumours (n = 7).

**Table 2:** Number of cases , age standardised (per 100 000) cancer incidence rates and number of person-years of observation for White & Indian children in Leicester, and for children in Mumbai & Ahmedabad, India. (All rates are standardised to the age distribution of the Segi standard population).

		Leicester				India			
		British Whites		British Indians		Mumbai		Ahmadabad	
ICC3/ICD code	Site	No.	ASR	No.	ASR	No.	ASR	No.	ASR
I/C70–72	CNS	7	1.6	9	4.2	263	1.7	55	0.9
II/C81–85	Lymphoma	3	0.6	6	2.8	176	0.7	63	0.5
III/C91–92	Leukaemia	11	2.6	11	5.8	472	3.1	135	2.2
IV/C22,C40–41,C49, C56,C62,C47,C74	Other solid tumours	17	4.0	7	3.5	436	2.8	116	1.9
*C22*	*Liver*	*1*		*0*					
*C40–41,C49*	*Bone, connective, soft tissue*	*8*		*4*					
*C56,C62*	*Gonadal*	*1*		*1*					
*C64*	*Kidney*	*5*		*1*					
*C47, C74*	*Neuroblastoma*	*2*		*1*					
	All sites	39	9.1	33	16.3	1453	9.3	413	6.7
Person-years of observation	436 021	207 281	15 625 574	6 225 080					

Table 3 displays the incidence rate ratios, adjusted for age, for Indian compared to White children for leukaemia, lymphoma, central nervous system tumours, all other solid tumours, and for all cancers combined. Leicester's Indian children had an increased risk of all cancers combined (RR = 1.82 (1.14 to 2.89); p = 0.01), with some evidence for an increased risk of leukaemia (RR = 2.20 (0.95 to 5.07); p = 0.07); lymphoma (RR = 3.96 (0.99 to 15.84); p = 0.04); and CNS tumours (RR = 2.70 (1.00 to 7.26); p = 0.05) relative to the White children. There was no difference in the incidence for the ‘all other solid tumours’ group (RR = 0.90 (0.37 to 2.18), p = 0.82).

**Table 3:** Rate Ratios (RRs) for cancers analysed for British White & British Indian children in Leicester.

ICC3/ICD code	Site	RR (95% CI)		p-value
I/C70–72	CNS	2.70	(1.00–7.26)	0.048
II/C81–85	Lymphoma	3.96	(0.99–15.84)	0.043
III/C91–92	Leukaemia	2.20	(0.95–5.07)	0.069
IV/C22,C40–41,C49, C56,C62,C47,C74	Other solid cancers	0.90	(0.37–2.18)	0.821
	All sites	1.82	(1.14–2.89)	0.013

### Sensitivity analysis

In the sensitivity analysis which assigned missing ethnicity values using multiple imputation, results for all cancer were unchanged (imputed RR = 1.82 (1.14–2.89).

### Comparison of British Indian children to children in India

Age standardised incidence rates in children in India from the Ahmedabad and Mumbai Cancer Registries for all cancers, and in the 4 cancer subgroups are also shown in Table 2.

The three most common cancers in the Indian registries were also leukaemia, lymphoma, and CNS tumours and age-standardised rates for Indian children in Leicester were higher than ASRs in the Indian registries for all cancers combined and in all the subgroups.

## Discussion

In this paper, we used self-assigned ethnicity to compare cancer incidence in Indian and White children in Leicester from 1996–2008. The age-standardised incidence rate ratios showed that Indian children had a higher risk for all cancers combined, with some evidence for an increased risk of leukaemia, lymphoma and CNS tumours. We also found that rates were higher in British Indian children compared to children in India.

Ethnicity refers to the group to which people belong as a result of certain shared characteristics, including geographical and ancestral origins, but particularly cultural traditions and languages [6]. Ethnicity therefore acts as a surrogate measure for both genetic and environmental/lifestyle exposures. It is not possible to distinguish between the two in this type of descriptive study but for most childhood cancers, it is likely that both are important.

Childhood leukaemia is thought to arise from early (in utero/post natal) genetic events and gene-environment interactions [24]. The prevalence of many of these exposures vary between ethnic groups. The higher incidence in British Indians may be due to their higher rates of consanguineous marriages (certainly amongst 1st generation migrants [25]) which may increase the risk of leukaemia in their offspring [26]. A maternal diet during pregnancy rich in topoisomerase II inhibitors has also been associated with an increased risk of childhood leukaemia [27] and, turmeric - a spice commonly used in Indian cuisine (through the action of its major active compound, curcumin) – acts as a topoisomerase II inhibitor [28]. Much of the focus of past epidemiological studies has been on exposures which may serve as proxies for childhood infection risk and immune protection such as daycare attendance and breastfeeding. British Indian children are more likely to be breastfed [29] and less likely to attend daycare [30].

The higher observed incidence of lymphoma in Indian children may be related to their much higher prevalence of Epstein Barr virus infection which has a strong association to certain subtypes of lymphomas [31].

The increased incidence of CNS tumours in British Indian children was unexpected and needs to be assessed in larger studies.

Whilst acknowledging the limitations of the data from cancer registries in India (see below), our results show that overall cancer incidence (as well as lymphoma, leukaemia, CNS tumours, and all other solid tumours) is higher among Indian children in Leicester than among those in India. This may give an indication of future childhood cancer incidence in India as it undergoes rapid development and urbanisation and with a third of India's population being less than 15 years of age, aetiological and prevention research is crucial as the cost of treatment is beyond the means of much of the population.

We are not aware of any studies of childhood cancer incidence in British Indians using self-assigned ethnicity. Due to the lack of ethnicity data, previous studies have tried to analyse all South Asian children together and have used different methodologies including non-population-based registers with analysis of relative frequencies of the different diagnostic groups [32]; survey based estimates of ethnic populations [33]; and name analysis software [12]. A more recent study in Yorkshire used a combination of name analysis, expert visual inspection, and HES ethnicity data but had a greater proportion of missing ethnicity data (24%) [11].

Although not directly comparable, these studies also found similar or increased incidence of all cancers combined in South Asian children compared to non-South Asians, as well as increased incidence of leukaemia and lymphoma but a similar or slightly lesser rate of CNS tumours.

The major strengths of this study (as discussed previously in our paper on adults [9]) are in the use of self-assigned ethnicity (the most accurate and reliable way of determining ethnicity [34]) which allows using the same ethnicity measure (self-assigned) for the numerator and denominator, and allows separation of South Asians into their individual ethnicities (Indian, Pakistani, Bangladeshi.) This method has various advantages over name analysis (which has been used in most previous studies due to lack of data on self-assigned ethnicity) where the numerator is estimated through name analysis, but the denominator through self-assigned ethnicity census data, leading to possible numerator/denominator mismatch. Misclassification error may also arise through (previously demonstrated) classification of South Asians as non-South Asians and vice versa (e.g. with the majority of Muslim names being derived from Arabic, and with more than 2500 [35] non-South Asian – White, Arab, North African, Iranian, Eastern European - Muslims in Leicester, it can be difficult to distinguish between these and South Asian Muslims through name recognition alone) [36]. Furthermore, name-analysis software groups all South Asians (Pakistani, Bangladeshi, Indian) together despite important differences in their diets and culture [37], and the comparison group (non-South Asian) is similarly a mix of different ethnic groups (White, Black, Chinese, etc.), whilst we were able to compare with British Whites only.

The main limitation of this study was the small number of cases and thus wide confidence intervals, and the study was underpowered to detect differences in individual tumour types or by age and sex subgroups. Numbers were also insufficient to adjust for income (which can be a potentially important confounder in studies of cancer and ethnicity [38]) although in Leicester levels of deprivation are similar in British Indians and Whites. Also, because this is a descriptive study we did not have individual level information on most exposures.

The comparison of rates between British Indian children and children in India is limited by the quality of data from Ahmedabad, and the fact that only about 20% of Mumbai's population is of Gujarati origin [23]. However, our finding that rates in British Indians are higher than both the ‘host country’ and country of origin is very unusual and is consistent with earlier suggestions that rates in India are underestimated due to under-diagnosis and under-ascertainment, particularly in rural areas [39].

## Conclusions

Despite advances in management of childhood haematological malignancies leading to improved survival rates, our aetiological knowledge remains limited. Given the consistent finding of increased risk of childhood leukaemia and lymphoma in South Asian and Indian children in England, there is a need for further epidemiological research to investigate possible risk factors. As the recording of self-assigned ethnicity in the HES database in other parts of the UK improves, it will be possible to analyse these differences by age and sex, as well as by subtypes of the childhood cancer diagnostic groups and to do a nationwide (and therefore better powered) comparison of incidence in Indians, Pakistanis and Bangladeshis. This will also help better determine the burden of disease and ensure appropriate provision of clinical services.
