# Correction: Childhood Cancer Incidence in British Indians & Whites in Leicester, 1996–2008

**DOI:** 10.1371/journal.pone.0098332

**Published:** 2014-05-20

**Authors:** The PLOS ONE Staff

There is an error in the second sentence of the "Classification of cancers" sub-heading under the "Patients and Methods" section. The correct sentence for this section is:

"We further arranged the cancers into 4 subgroups (leukaemia, lymphoma, CNS tumours, all other solid tumours) based upon the International Classification of Childhood Cancer (ICD-10) system [18], corresponding respectively to its diagnostic groups I, II, III and IV–XII, as used in previous studies [11]."
